# Characterization of Critical Amino Acids in the Transport and Selectivity of the Plant Na^+^/H^+^ Exchanger Plasma Membrane SOS1

**DOI:** 10.3390/ijms26083518

**Published:** 2025-04-09

**Authors:** Asad Ullah, Debajyoti Dutta, Larry Fliegel

**Affiliations:** 1 Department of Biochemistry, University of Alberta, Edmonton, AB T6G 2R3, Canada; asad1@ualberta.ca; 2 Department of Biotechnology, Thapar Institute of Engineering and Technology, Patiala 147004, Punjab, India; debajyoti.47@gmail.com

**Keywords:** *Arabidopsis thaliana*, ion transport, membrane protein, Na^+^/H^+^ antiporter, salt tolerance, SOS1

## Abstract

SOS1 is a Na^+^/H^+^ antiporter found in the plant membrane of *Arabidopsis thaliana* and serves as a major transporter that extrudes Na^+^ across the plasma membrane of cells in exchange for intracellular H^+^. The first 450 amino acids comprise the membrane transport domain. Using a yeast heterologous expression system, we examined nine different mutations that may either change specificity or improve salt tolerance. E261K had minor negative effects on the ability to confer tolerance to LiCl and NaCl. Mutation A399V had minor effects, lowering LiCl tolerance and slightly improving NaCl tolerance, as did the double mutant E261KA399V. Four different mutations of amino acid Y346 had varying effects. The Y346R mutation resulted in a major improvement in LiCl tolerance but did not affect NaCl tolerance. The L375I mutant showed impaired NaCl tolerance, whereas the Q362L mutant exhibited minor effects on salt tolerance. Our results demonstrate that amino acid Y346 is critical in ion selectivity and its mutation can dramatically improve LiCl salt tolerance. Other mutations showed minor improvements in the ability to confer NaCl tolerance (Y346F, A399V, and Y346A), leaving open the possibility that such mutations might improve salt tolerance in intact plant species.

## 1. Introduction

Most plants including crop plants, are highly sensitive to salt stress. Salt stress causes osmotic stress and water deficit which result in biochemical problems affecting plant growth rate and survival [1,2,3].

Plants usually maintain high intracellular K^+^ (100–200 mM) and low Na^+^ (1–10 mM). For crop plants, soil salinity is a key factor that limits plant growth and agricultural productivity [4].

The abiotic stresses of drought and salinity can lead to reduced plant growth, wilting, or death. These stresses can act through protein denaturation, aggregation, and inhibition of protein synthesis. During the reproductive stage, abiotic stress can result in flower drop, pollen tube deformation, ovule abortion, pollen sterility, and reduced yield [5]. Under drought stress, crop development exacerbates the issue of extreme water use in agriculture. Turgor pressure is decreased, which is a delicate physiological mechanism that regulates cell growth [6].

Plants respond to abiotic stress in several ways. Genes such as WKRY transcription factors, including SIWRKY8, GmWRKY12, MxWRKY53, and MfWRKY40, upregulate plant tolerance to drought and salinity by increasing the levels of stress-response genes. They act through various mechanisms, including maintaining water content [7,8,9,10,11,12,13,14]. Other transcription factors that play important roles in plant responses to drought and salinity include CBF (C-repeat binding factor) transcription factors, such as DREB1/CBF [15,16], and MYB transcription factors [16,17]. Both are involved in cellular stress responses. ERF (ethylene responsive factor) [18] and bHLH (basic helix-loop-helix) transcription factors [19,20,21] also play significant roles in plant responses to salinity and drought, activating stress-mitigating responses. NAS (nicotianamine synthase) genes, such as MxNAS3 [22], additionally play a role in stress, drought, and salt tolerance, as do several other transcription factors, including MbMYBC1 [23], MbICE1 [24], FvMYB114 [25], and FvMYB44 [26].

**Figure 1:**
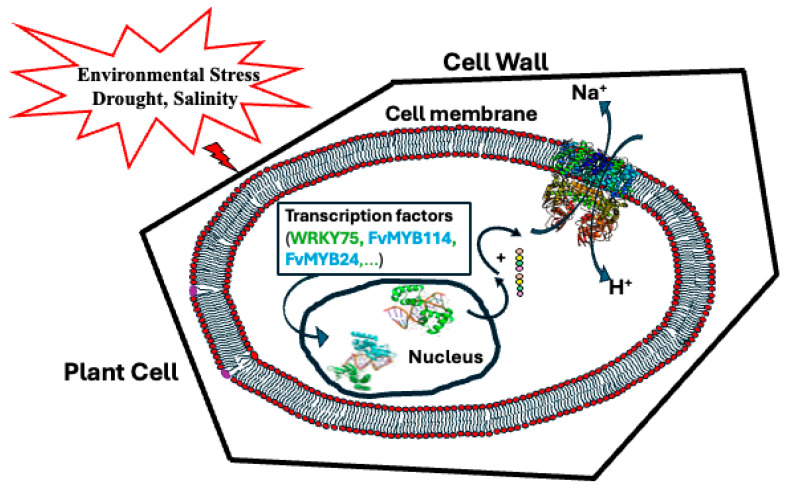
Schematic diagram of the activation of the *Arabidopsis thaliana* (At) SOS1 protein in response to environmental stress. Environmental stresses, such as drought and elevated salinity, lead to an increase in the levels of certain transcription factors, which results in elevated production of SOS1. This, in turn, improves salt tolerance and maintains cellular ion balance. (From [23,24,25,26,31,32,33,34,35,36], and see text for further discussion).

One important mechanism that plants use to deal with Na^+^ stress is the signaling pathway known as the Salt Overlay Sensitive (SOS) pathway, which detects salt stress and takes protective measures [27]. An important component of the SOS pathway is SOS1, the sodium transporter of the pathway. It is a plasma membrane Na^+^/H^+^ exchanger that exchanges an extracellular H^+^ for an intracellular Na^+^ ion. It therefore uses the proton gradient generated by the plasma membrane H^+^-ATPase to remove intracellular sodium [28]. Plasma membrane SOS1 is chiefly expressed in the root and xylem of plants [28]. SOS1 can also be expressed in vacuoles, where it contributes to salt tolerance [29,30]. It has been demonstrated in a number of plant types, including *Arabidopsis thaliana*, that SOS1 is upregulated in response to salt stress by several transcription factors (Figure 1), improving the plant’s ability to cope with salt stress [31,32,33,34,35]. By promoting ion transport, cellular ion balance is maintained [33,36].

Other types of cation/H^+^ antiporters are present in plants including intracellular Na^+^/H^+^ antiporters and K^+^(Na^+^)/H^+^ antiporters which also assist in coping with salt stress [37,38]. However, SOS1 is of special interest because of its clear role in permitting salt tolerance in many plant species [39,40,41,42,43,44,45] without a requirement for vesicle trafficking. The overexpression of SOS1 improves salt tolerance in plants including in crop plants [45,46,47,48]. Similarly, enhancing SOS1 activity through regulatory pathways in plants promotes salt tolerance [49]. Conversely, decreased SOS1 expression leads to loss of salt tolerance in different species [50,51,52,53]. *At*SOS1 (*Arabidopsis thaliana* SOS1) can confer salt tolerance across distant species. *Schizosaccharomyces pombe* (*S. pombe*) has one principal plasma membrane salt tolerance protein, and its deletion results in a salt-sensitive yeast *S. pombe* strain [54]. The Sod2-deficient strain shows improved salt tolerance when *At*SOS1 is expressed in these yeast [55,56] making it an excellent model system for examining the effects of mutations on the protein.

Given the importance of SOS1, we thought it essential to investigate the amino acids crucial to its function. In addition, we noted that some amino acid residues have been suggested to enhance or be critical in SOS1 activity. For example, Quintero [57] showed that mutations E261K and A399V enhanced the activity of SOS1 introduced into *Saccharomyces cerevisiae*. A different study [58] examined how selection pressure acted on the SOS1 gene in the *Populus* genus to mediate saline tolerance. They found several amino acid sites selected for in salt tolerantsalt-tolerant plant types, including amino acids 345, 361, and 374. In the present study, we examined and further characterized the mutation of the corresponding amino acids in a different system, in the salt-sensitive yeast strain *S. pombe.* This species has its sole plasma membrane salt tolerance protein sod2 deleted [54]. Earlier [56] we demonstrated that we could express *Arabidopsis thaliana* SOS1 functionally in this system using a shortened version of *At*SOS1. In this study, we used this construct to focus on effects mediated by the more conserved membrane transport domain, as opposed to those on the regulatory domain. We characterize the effects of specific mutations implicated in improved salt tolerance using the shortened protein. We examine effects on expression, alterations in different cation transport, and examine some combinations of mutations. Our results demonstrate that some of these mutations improved salt tolerance in the SOS1 (short) protein, which coded for the membrane domain without the regulatory tail. We also found that Y346 was an amino acid important in the specificity of the SOS1 protein, suggesting it may be a critical component of the ion coordination during transport.

## 2. Results

### 2.1. SOS1 Alignment and Modeling

Salt-tolerant plant strains have evolved adaptations to cope with the presence of extraneous sodium. Meng and Wu [58] conducted a systematic examination of some of these adaptations in SOS1 in plants subjected to selection for salt tolerance. The Turanga group of plants underwent selection for drought and salinization in other areas where plants are nearly unable to grow. In the genus Populus, some species in particular have undergone selection pressure for salt tolerance, such as *Populus euphratica*. Several amino acids were changed under selection pressure in *Populus euphratica*, including R345, Leu361, and Ile374.

**Figure 2:**
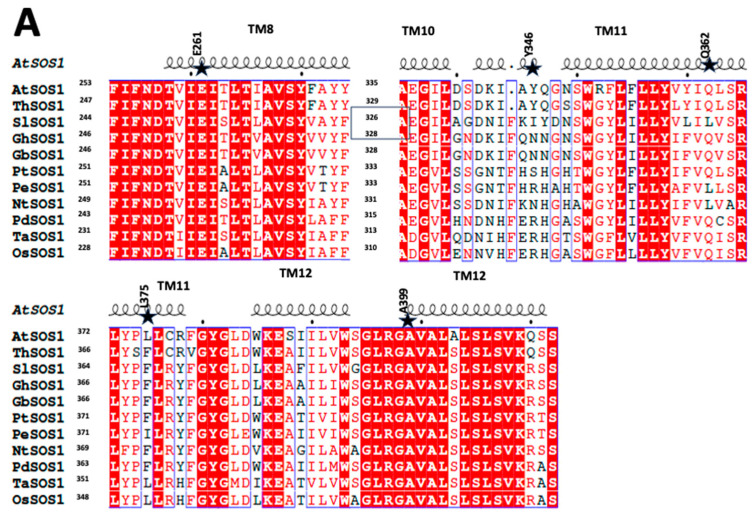

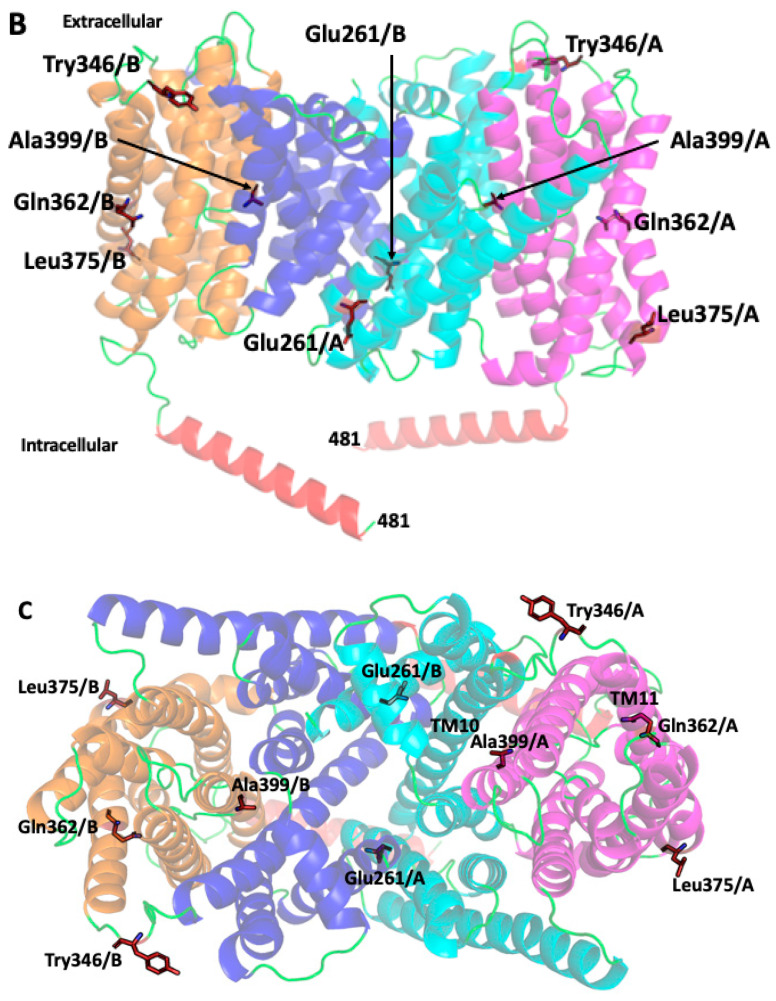
Analysis of *Arabidopsis thaliana* (At) SOS1 protein. (**A**) Multiple sequence alignment of plant SOS1 demonstrating the segments harboring *A. thaliana* SOS1 amino acids investigated in this manuscript. The same amino acids are shown using an asterisk. The red background white colored amino acids are conserved amino acids. The red-colored amino acids are those of similar types. TM# denotes the transmembrane segments. The coils on the top of the alignment represent the helices of the *A. thaliana* SOS1 structure. Amino acid numbers are indicated at the left and are aligned with the Arabidopsis thaliana SOS1 sequence, beginning at amino acids 253, 335 and 372. At, *Arabidopsis thaliana* (NP_178307.2); Th, *Thellungiella halophila* (ABN04857.1); Sl, *Solanum lycopersic*um (NP_001234698.2); Gh, *Gossypium hirsutum* (AMY98958.1); Gb, *Gossypium barbadense* (A0A5J5SE13); Pt, *Populus trichocarpa* (UniRef100_U5G5K6); Pe, *Populus euphratica* (DQ517530); Nt, *Nitraria tangutorum* (AGW30210.1); Pd, *Phoenix dactylifera* (XP_008798100.1); Ta, *Triticum aestivum* (Q4L224); Os, *Oryza sativa* (Q5ICN3). The red background represents conserved residues. The red-colored residues are similar types of amino acids. The residues being investigated are highlighted with an asterisk. The transmembrane segments corresponding to a particular amino acid sequence are listed at the top. The square box indicates the second segment of the alignment. The only SOS1 polypeptide segments shown are those harboring the amino acids considered in this work. (**B**,**C**), Cryogenic electron microscopy structure of the *At*SOS1s (amino acid 32–481) dimer (modified from the PDB 7Y3E) [59,60]. The N-terminal residues (1–31) are not shown for clarity. Two different color sets are used to refer to each monomer of the dimer chain A (magenta and cyan) and chain B (orange and blue). The dimerization domains (cyan and blue) and transport domains (magenta and orange) are highlighted. (**B**) view of *At*SOS1 parallel to the membrane plane. (**C**) view of *At*SOS1 perpendicular to the membrane from the extracellular side.

In our study, we compared the sequence of several species including that of *Populus euphratica* and *Arabidopsis thaliana* SOS1 (Figure 2A). Amino acids in Arabidopsis thaliana SOS1 that correspond to often changed amino acids in salt-tolerant species were Tyr346, Gln362, and Leu375. This led us to mutate these amino acids in *At*SOS1 (as shown below). Figure 2 shows that in *Populus euphratica*, the residue equivalent to Tyr346 of *Arabidopsis thaliana* SOS1 is an Arg; the residue in *Populus euphratica* equivalent to Gln362 of *Arabidopsis thaliana* SOS1 is a Leu; and the residue in *Populus euphratica* equivalent to Leu375 is an Ile. These changes were investigated in the present study. Additionally, the mutations E261K and A399V have been previously shown to enhance the activity of SOS1 when introduced into *Saccharomyces cerevisiae* [57]. Therefore, this group of amino acids was chosen for analysis in further studies, and this corresponding region is shown in Figure 2A. The residues corresponding to E261 and A399 of *At*SOS1 are tightly conserved.

SOS1 was examined in earlier studies using cryo-electron microscopy [61]; however, that analysis was not detailed enough to reveal the protein’s structure. We recently used multiple sequence analysis to predict a topology of 13 transmembrane segments [56]. However, more recently [59,60] the detailed structure of SOS1 has been determined. We therefore used this structure (Figure 2B,C) to illustrate the locations of the residues that we examined in this study. Analyses of the cryo-EM structure of *A. thaliana* SOS1 reveal that Tyr346 is located in the extracellular loop connecting transmembrane helix 10 (TM10) and transmembrane helix 11 (TM11) (Figure 2B,C). TM10 is part of the dimerization domain of the protein, while TM11 belongs to the transport domain [59,60]. Multiple-sequence alignment (Figure 2) shows that residues between these two helices are not conserved. However, the loop maintains a critical balance between negatively and positively charged residues. Multiple-sequence [62]. Further multiple sequence alignment across a wide range of plant SOS1 proteins indicates that, in most plants, a negatively charged amino acid is paired with a positively charged residue in this loop (Figure 2A). Interestingly, in *Populus euphratica* SOS1, a salt-tolerant species, the corresponding position of Tyr346 in *A. thaliana* is occupied by arginine, and the same loop lacks any negatively charged residues. Given *P. euphratica*’s tolerance to salinity, we attempted to replace Tyr346 of *A. thaliana* SOS1 with arginine to explore its functional implications. Additionally, the cryo-EM structure and sequence of *Oryza sativa* SOS1 (OsSOS1), available in the PDB database, show that its TM10-TM11 loop contains an arginine residue preceded by an aspartate in the polypeptide sequence (Figure 2).

**Figure 3:**
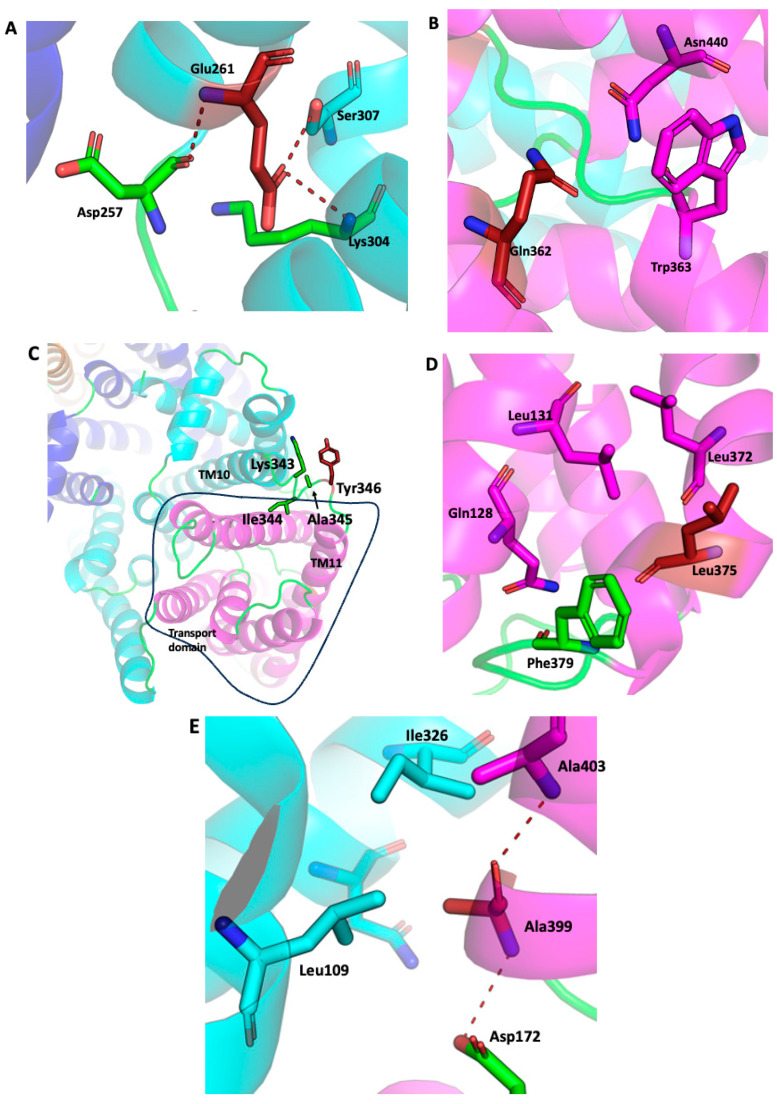
Close-up view of the residues being investigated. *A. thaliana* SOS1 (PDB 7Y3E) is used for the representation. The mutated residues are shown in firebrick color. Residues from the loop are shown in green, where the magenta and cyan colors represent transport and dimerization domains, respectively. (**A**) Glu261 and its interacting distance with the Lys304 and Ser307. (**B**) Proximity of Gln362 close to Trp363 and Asn440. (**C**) Tyr346 is shown to reside on the extracellular loop joining the TM10 and TM11. TM10 and TM11 are the parts of the dimerization domain and the transport domain, respectively. In the polypeptide chain, Tyr346 is preceded by the residues Lys343, Ile344, and Ala345. (**D**) Leu375’s side chain is surrounded by hydrophobic side residues such as Leu131, Leu372, and Phe379. (**E**) Ala399 is located at the center of the protein and is located at the discontinued helices of the transport domain.

Figure 2 illustrates close-ups of the residues investigated in this work. The Glu261 interaction network is shown in Figure 3A. Glu261 is located at the intracellular side of the dimerization interface. This acidic residue closely interacts with Asp257 through a backbone interaction, whereas it interacts with Ser307 through a side-chain interaction and with Lys304 through a side-chain and backbone interaction. Figure 3B shows the proximity of Glu362 to the discontinues helices and proximity to Trp363 and Asn440. Figure 3C illustrates the extracellular location of Tyr346 in the loop joining TM10 with TM11. The hydrophobic network provided by the residue Leu375 together with Leu131, Leu372, and Phe379 is shown in Figure 3D. The amino acid Ala399 and its juxtaposition to Ile326 and Leu109 in the center of the protein provide a hydrophobic groove and are shown in Figure 3E.

### 2.2. Expression of Wild Type and Mutant SOS1 in S. pombe

**Figure 4:**
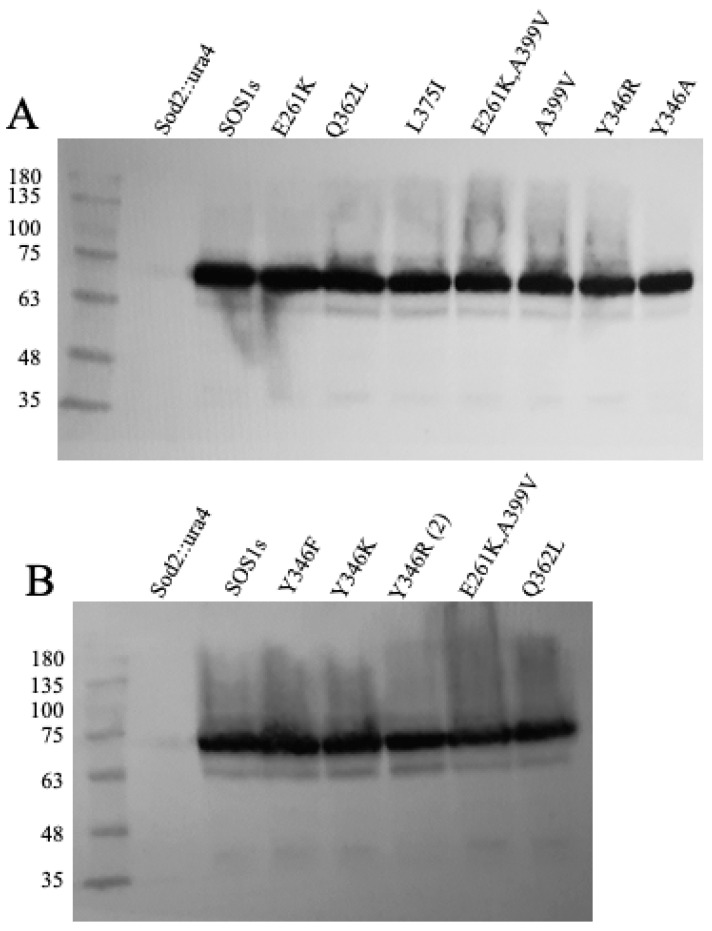
Expression of various length SOS1 proteins. (**A**,**B**) Western blot analysis of expression of SOS1s proteins. Equal amounts of cell extracts from *S. pombe* strains expressing various SOS1s-GFP constructs were blotted with anti-GFP antibody as described in Section 5. SOS1s is the wild-type (shortened) protein, and Sod2Ura4 is the knockout strain. Other lanes are SOS1s protein with the indicated mutation. (Y346(2) indicates a second sample of this cell extract.).

To study the amino acids critical in SOS1 function, we expressed the protein in yeast in a knock-out strain (sod2::ura4) deficient in endogenous salt-tolerant protein as described earlier [63,64,65]. To confirm that both wild-type and mutant SOS1 proteins were expressed, we used Western blotting against the GFP tag present on the SOS1 protein C-terminal end. The results (Figure 4) demonstrate that all the mutants and the wild-type SOS1s proteins were expressed at similar levels. Mutation of SOS1s did not appear to adversely affect expression levels. The size of the shortened SOS1 protein was the same as that reported earlier [56].

### 2.3. Salt Tolerance of Wild Type and Mutant SOS1 in S. pombe

**Figure 5:**
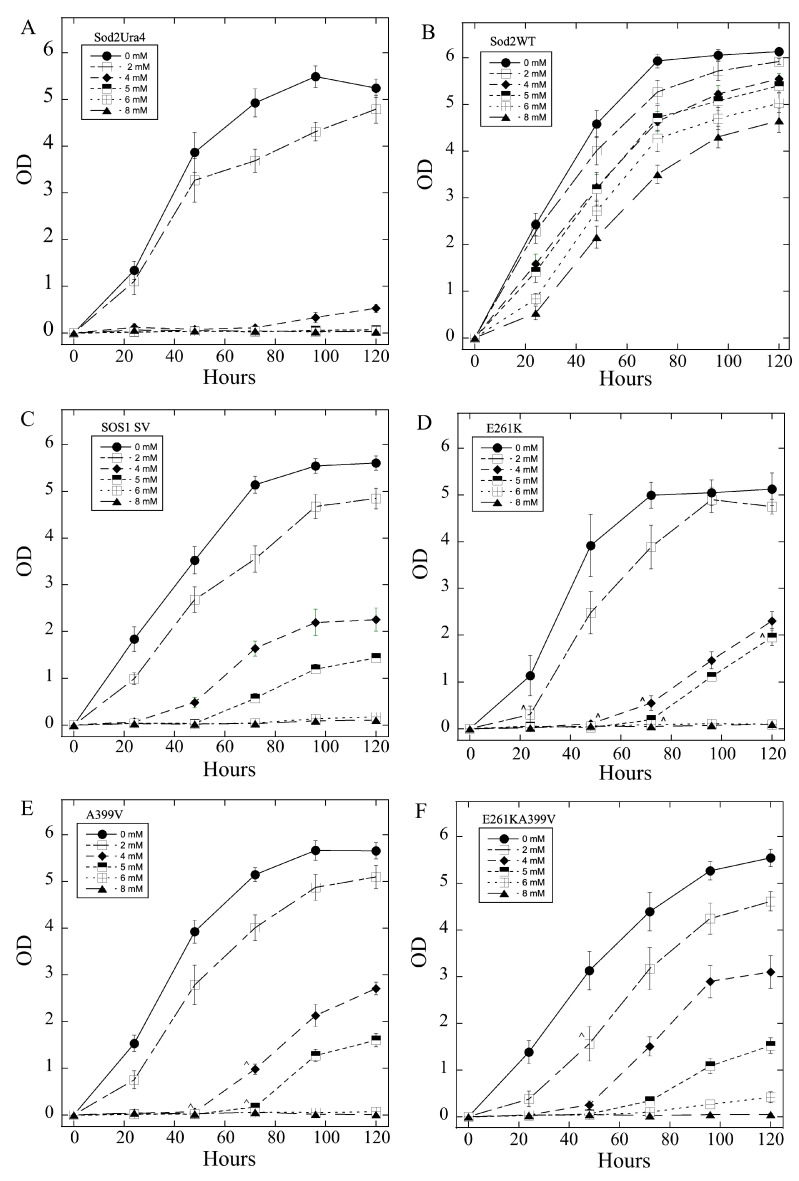

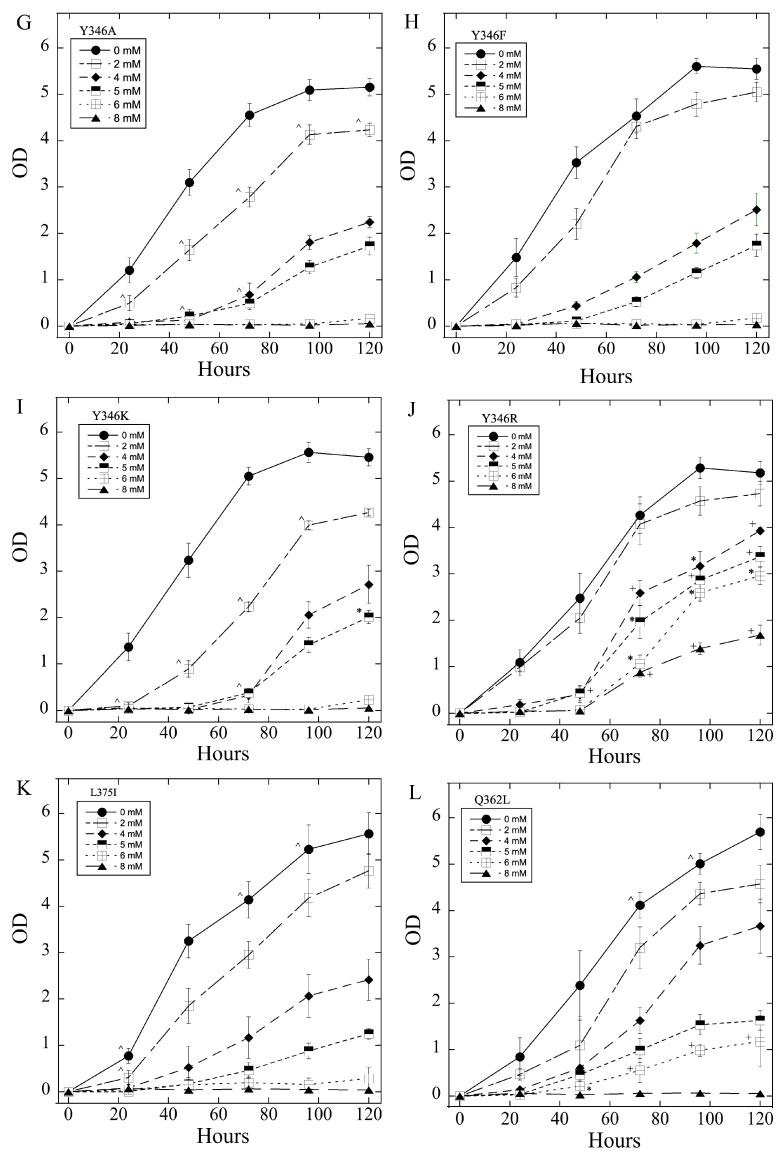

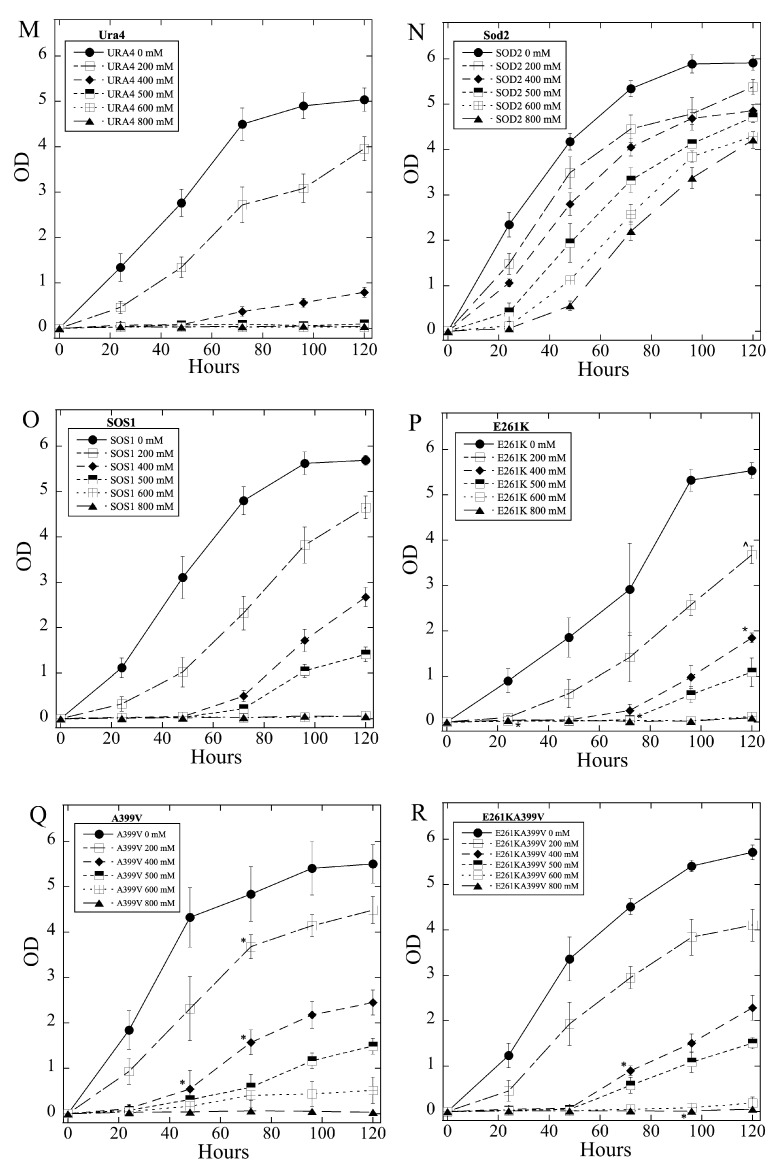

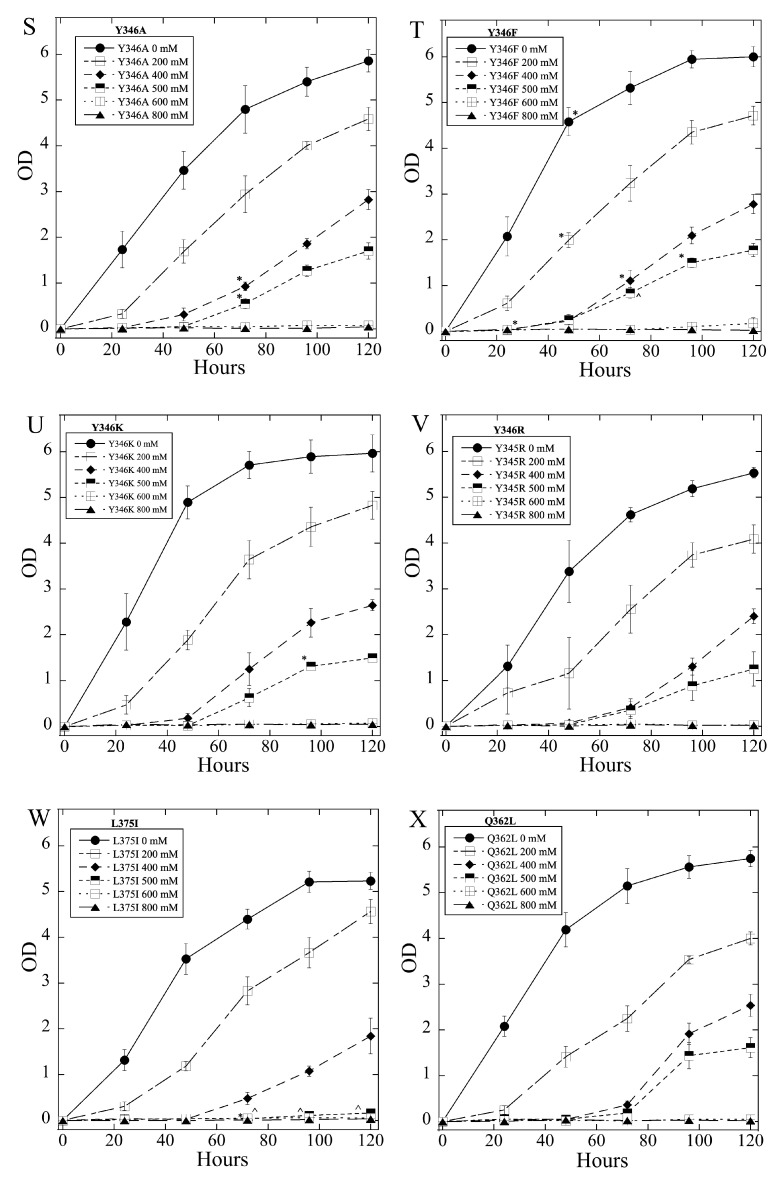
Growth in liquid medium of sod2 knockout *S. pombe* and the same strain containing either wild-type sod2 or SOS1s protein or SOS1s mutant proteins. LiCl (**A**–**L**) or NaCl (**M**–**X**) tolerance of the strains was assessed by growing 2 × 10^6^ of inoculated cells in 2.5 mL of medium at 30 °C for up to 120 h. Growth was assessed by measuring the absorbance of the cell suspensions at 600 nm at the times indicated. Results are the mean +/− SE of at least three determinations. *S. pombe* were grown in the presence of 0, 2, 4, 5, 6, or 8 mM LiCl or 0, 0.2, 0.4, 0.5, 0.6, or 0.8 M NaCl as indicated. (**A**,**M**) growth rates of control, *Sod2Ura4* cells. *Sod2Ura4* refers to *S. pombe* with the sod2 knockout described earlier [36]. (**B**,**N**) *S. pombe* containing wild-type sod2 and (**C**,**O**) *S. pombe* containing wild-type SOS1s. (**B**) as in A, except in various LiCl-containing media as indicated. Other growth curves (**D**–**L**,**P**–**X**) are for yeast containing SOS1s with the indicated mutations. *, ^ significantly different from SOS1s mutant at *p* < 0.05 or 0.01, respectively.

We next examined the ability of wild-type SOS1 and SOS1 mutants, along with sod2, to restore salt tolerance in *S. pombe* that has endogenous sod2 deleted. LiCl and NaCl are both transported by sod2 and a variety of other Na^+^/H^+^ exchangers [64]. LiCl was used for assays in liquid media since it is toxic at lower concentrations, avoiding the osmotic challenge of high concentrations of NaCl. Li also has a smaller ionic radius than Na (0.76 A vs. 1.02 A) [66]. Figure 5 panels A-X show the growth of various *S. pombe* strains containing the indicated mutant. Panels A-L illustrate growth in LiCl of varying concentrations and panels M-X in NaCl of varying concentrations. Table 1 summarizes the results of the experiments.

The growth of the knockout strain (Figure 5A) occurred in the presence of 0 and 2 mM LiCl. The positive control, the yeast Na^+^/H^+^ exchanger sod2 conferred tolerance to all concentrations of LiCl tested, up to 8 mM. The SOS1 constructs conferred partial tolerance to LiCl, allowing growth in up to 4 and 5 mM LiCl (Figure 5C). Growth of the mutants shown in Figure 5D–L (mutants E261K, A399V, E261KA399V, Y346A, Y346F, Y346K, L375I, and Q362L) in LiCl was similar to the wild-type SOS1, but varied in interesting and sometimes significant ways. For SOS1 with the E261K mutation (Figure 5D), the general pattern of growth was similar, but growth at the lower LiCl concentrations (2, 4, and 5 mM) was reduced compared to the controls at some time points. In contrast, with the E261K mutation, there was a slight improvement in growth at some intermediate time points in 6 and 8 mM LiCl, though these changes were quite small. SOS1 with the A399V mutation (Figure 5E) conferred similar salt tolerance as the wild-type protein, with only minor changes. In 4 and 5 mM LiCl, growth was somewhat reduced. The E261KA399V mutant (Figure 5F) displayed changes similar to the A399V mutant, with slightly reduced growth in lower LiCl concentrations. Both the Y346A (Figure 5G) and Y346K (Figure 5I) mutant proteins exhibited substantially reduced salt tolerance in lower LiCl concentrations, although the Y346K mutant was also somewhat ineffective in conferring salt tolerance at higher LiCl concentrations. In contrast to these two mutations, another mutation at this position, Y346F (Figure 5H), had no effect on LiCl tolerance. The greatest effect of any mutation was seen with the Y346R mutation (Figure 5J), which showed a remarkable improvement in LiCl tolerance at both high and low LiCl concentrations. This mutation greatly improved LiCl tolerance over a wide range of concentrations and times. The mutations L375I and Q362L (Figure 5K,L) did not significantly affect LiCl tolerance. The Q362L mutation had a positive effect on growth in 6 mM LiCl, but both mutations slightly decreased growth in the absence of LiCl during exponential growth. However, the final growth stage reached in the absence of LiCl have a was not different from cells with wild-type SOS1.

Tests of NaCl tolerance are shown in Figure 5M–X and summarized in Table 1. The growth of the sod2 knockout strain (Figure 5M) occurred in 0 and 200 mM NaCl with some minimal growth in 400 mM NaCl. Yeast containing the wild-type sod2 protein grew robustly robustly in the presence of 200 to 800 mM NaCl (Figure 5N). Expression of the SOS1s (Figure 5O) protein improved salt tolerance greatly in 400 and 500 mM NaCl but not in 600 mM NaCl or 800 mM. There were varying effects of mutations in SOS1s on NaCl tolerance, mostly smaller changes. The E261K (Figure 5P, Table 1) mutation inhibited NaCl tolerance somewhat in lower NaCl concentrations (200–500 mM) at the later stages of growth. The mutations A399V, E261KA399V, and Y346A all had slight but significant positive effects on salt tolerance in lower NaCl concentrations (200–500 mM). These were in the middle stages of the growth curve. The mutation of Y346 to Phe (Figure 5T) also had some positive effects in lower concentrations of NaCl and there was also a slight but significant improvement in growth relative to the control, in the absence of NaCl. Other mutations of Y346 had either no effect on growth (Y346R, Figure 5V) or a minor but significant positive effect on growth by mutation with the Y346K mutation (Figure 5U) in 500 mM NaCl. The L375I mutant (Figure 5W) exhibited only negative effects on growth. Cells with SOS1s carrying this mutation showed NaCl tolerance similar to the knockout in 500 mM NaCl. The Q362L mutation did not change growth in NaCl-containing liquid media.

**Figure 6:**
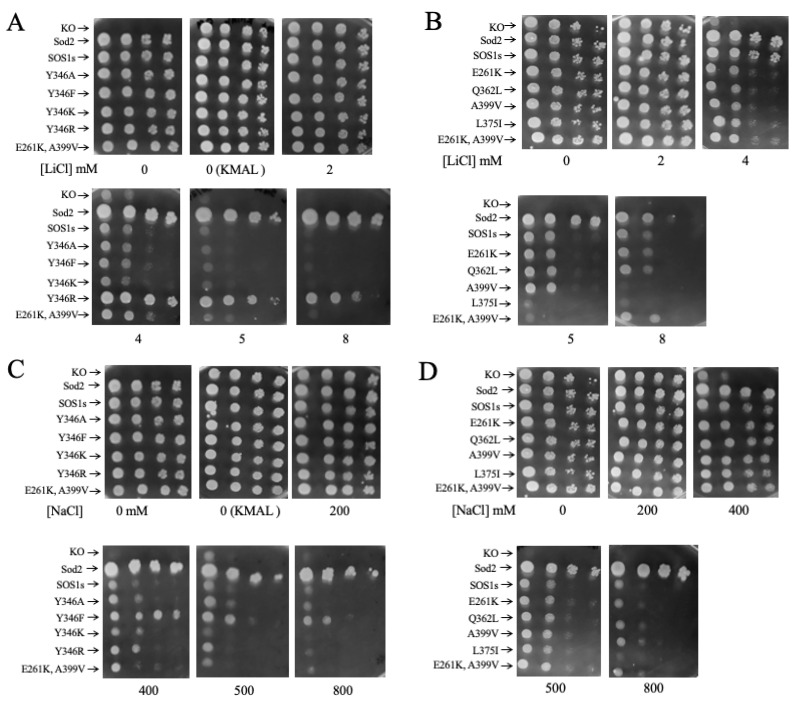
Growth of wild-type (WT) and mutant SOS1-containing *S. pombe* on solid media in the presence or absence of varying amounts of LiCl (**A**,**B**) or NaCl (**C**,**D**). Samples of the various strains were obtained from stationary-phase cultures that were serially diluted 1:10 repeatedly. They were spotted onto minimal media plates with the indicated concentrations of NaCl or LiCl. Plates were incubated for 72 h at 30 °C as described earlier [34]. *S. pombe* containing wild-type Sod2 was used as a positive control. The results are typical of 3 repeats. KO, *S. pombe* containing the wild-type sod2 knocked out as a negative control. ((**C**), top left, shows growth on KMA plate without Leu. All other growth was performed on KMA media plus Leu).

We next (Figure 6, Table 1) examined salt tolerance in the mutants on solid media using techniques we have described earlier [62] in order to verify results seen in liquid media. Figure 6A,B illustrate the growth in different concentrations of LiCl and Figure 6C,D in different concentrations of NaCl. In LiCl and in NaCl, the wild-type sod2 protein permitted robust growth in all concentrations tested. *S. pombe* with knock out of the sod2 protein grew well in up to 2 mM LiCl and 200 mM NaCl but not at higher concentrations. The wild-type SOS1 protein improved growth in 400 and 500 mM NaCl and 4 and 5 mM LiCl but growth was still reduced compared to lower cation concentrations. These results mirrored those of the growth curves in liquid media with only some minor differences where there were subtle changes in salt tolerance (Table 1). The various mutations of amino acid Y346 were largely similar to that of wild-type SOS1, with the notable exception of yeast with SOS1 with the Y346R mutation, which improved growth greatly in up to 8 mM LiCl. In both liquid and solid media, there were also smaller improvements in NaCl tolerance with the Y346F mutation. Mutations of A399V and the double mutant cause impaired growth the presence of LiCl. Other cells containing mutations of the SOS1 protein did not exhibit significant changes in cation tolerance on solid-phase media, with one minor exception. In the case of the L375I mutant, growth was inhibited at higher LiCl concentrations.

**Figure 7:**
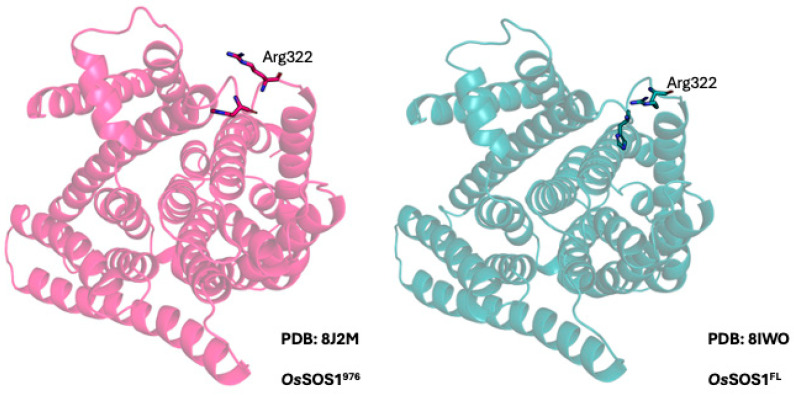
Comparison of the structures of *Oryza sativa* SOS1 (residues up to 976) (OsSOS1) PDB:8J2M and full length *Oryza sativa* SOS1 PDB:8IWO [59]. The structure reveals the flexibility of the TM10–TM11 loop harboring the residue Arg322, which is present at the corresponding positions of *Arabidopsis thaliana* SOS1 Try346.

Because we found extremely interesting effects when we mutated amino acid Y346 of *Arabidopsis thaliana* SOS1, we examined the structure in the region of Arg322 of *Oryza sativa* SOS1 (the truncated rice Na^+^/H^+^ exchanger). This amino acid residue of *Oryza sativa* SOS1 corresponds to Y346 in *Arabidopsis thaliana* SOS1 (Figure 2). Figure 7 illustrates two different structures of the TM10-TM11 loop that contains Arg322. Both structures show that the loop is comparatively flexible and contains at least two positively charged residues and one negatively charged residue (Figure 7). The different positions of Arg322 indicate that there is flexibility in *Oryza sativa* SOS1 TM10-TM11 loop harboring the Arginine residue.

## 3. Discussion

Soil salinity is a key factor limiting plant growth and agricultural productivity [4]. As noted above, SOS1 is a key transporter involved in salt tolerance in plants and it extrudes an intracellular Na^+^ ion in exchange for an extracellular H^+^ [27]. It is obviously desirable to enhance SOS1 activity in this regard to enhance salt tolerance, plant growth SOS1 and agricultural activity. Knowledge of the transport mechanism and critical amino acids involved in transport would undoubtedly be useful in this regard. SOS1 of Arabidopsis was used as a model system in this study and it has a very large internal regulatory tail. Earlier we [49] examined the effects of overexpression of SOS1 cytosolic tail fragments which was believed to lead to sequestration of inhibitory proteins and elevation of SOS1 activity, with a resultant increase in salt tolerance. Others have shown in a several plant species, that overexpression of SOS1 or SOS1-like proteins can result in elevated salt tolerance and deletion results in salt sensitivity [46,48,67]. However, in addition to other regulatory proteins, SOS1 contains a regulatory tail with regions inhibitory to activity and this can confound effects of simple overexpression of the protein [48]. Also, the carboxyl terminal regulatory region is more variable between species and subject to many regulatory factors which are likely to be variable from one plant species to another. Our goal in this study was to characterize ion transport itself in the membrane domain including an examination of amino acids involved in Na^+^ vs. Li^+^ transport. Additionally, we attempted to identify putative changes in the SOS1 protein that might improve transport and might be widely applicable in different species. We therefore studied the membrane domain which is more conserved and responsible for cation binding and transport and can mediate salt tolerance in yeast [56,68].

We have previously developed an expression and mutation system for *S. pombe* [56]. In this species elimination of only one salt tolerance protein, sod2, results in salt sensitivity and SOS1 has earlier been shown to restore salt tolerance after the removal of sod2 [48,56,69]. We have also earlier shown that a shortened version of SOS1, without the inhibitory/regulatory domain is functional in this system [56]. We therefore used this system in the present study, to examine the effects of several amino acids that were suggested to be important in salt tolerance. One group of such amino acids we examined was amino acids Tyr346, Gln362 and Leu375. A phylogenetic analysis had earlier suggested that the corresponding amino acids were important in salt tolerance in *Populus euphratica* [58]. Figure 2A shows a comparison of amino acids 314–393 of Arabidopsis thaliana SOS1 with the corresponding sequence of *Populus euphratica* and other related species. Amino acids mutated in this study, Tyr346, Gln362 and Leu375, are indicated and correspond to those suggested to be important in salt tolerance in *Populus euphratica*. Another pair of amino acids E261 and A399 had earlier [57] also been suggested to be significant in mediating salt tolerance and we examined these and also attempted to examine the effect of inserting a pair of putative salt improving mutations in the same system, in an attempt to further increase salt tolerance (Table 1).

Results with amino acids E261 and A399 were varied. Results with liquid media were more easily quantified. Results with solid media are less sensitive or quantifiable, but they generally agreed with those found in liquid media notably agreeing with the larger more easily viewed changes. In our system, the individual mutation of E261K did not result in an improvement in tolerance to NaCl but did result in a minor improvement in LiCl tolerance in liquid media. There was also a minor reduction in NaCl tolerance. This is in contrast to results found earlier [57] and we can only attribute this to the different system we used. In the case of the A399V mutation, this did result in an improvement in NaCl tolerance. Thus, we can state that there was a minor effect of this mutation which agrees with the earlier report [57] but the effect was not large. It seems as though this mutation is a more robust one which might be favored for further studies in an intact plant model. The combined E261K/A399V mutations showed an effect similar to that of the A399V mutation alone in liquid media, so there was no additional advantage of adding the A399V mutation to the mutant E261K. Why the earlier report showed greater effects than our study is not clear, though this may be due to either the different system used, or due to the absence of the regulatory tail. Whatever the cause, the potency of the mutations effects was not easily transferred to our system. This might mean that they are not easily transferrable to other plant systems. However, further experiments are necessary to determine this.

The Ala399 amino acid is on TM12 and is in the unwound region mid membrane which is a part of the characteristic Na^+^/H^+^ exchanger fold of SOS1 (Figure 3E). The fold has been shown to be critical to Na^+^/H^+^ exchanger activity [70]. Thus, it seems reasonable that a mutation in this fold might affect transport. We found mixed results with this amino acid and how this improved transport in an earlier study is not yet clear [57]. In contrast Glu261 is present on the N-terminal of TM11 close to the cytoplasmic side of the protein near amino acids Lys304 and Ser307 (Figure 3A). There it could play a part in the attraction of cations to the transport core. It is difficult to understand how a change from a negatively charged glutamic acid to a positively charged lysine can enhance the transport of LiCl. Li has a smaller ionic radius than Na, so perhaps a change to the positive charge excluded this amino acid from playing a role in electrostatic environment mediating cation coordination, allowing the balance of negatively charged residues to release Li more quickly. Alternatively, removing one point of coordination may more suitably coordinate this smaller ion, in comparison to Na^+^. These theories have yet to be tested.

Mutation of amino acid Q362 to leucine did have a positive effect on growth in higher concentrations of LiCl in liquid media. Q362 is found in relatively close proximity (Figure 2 and Figure 3B) to the NhaA fold [70] that is critical in cation transport. Thus, it is possible that it affects cation coordination, an effect more evident at higher concentrations. Since this was not found with NaCl, and only at higher LiCl concentrations, it is likely that the effect is a change in cation coordination rather than an enhancement of the turnover rate.

The mutation of L375 to isoleucine had a pronounced negative effect on growth in liquid medium containing NaCl at various concentrations. This is in contrast to a previous report [58] that suggested that the corresponding mutation in *Poplus* enhances salt tolerance. We suggest that the beneficial effect of changing this amino acid to isoleucine may be species-specific, and not easily transferrable to the SOS1 protein of *Arabidopsis thaliana*. This makes it a less desirable target as a method of increasing salt tolerance in many plant species.

The most interesting effect we found was with mutation of amino acid Y346. As noted above, the corresponding amino acid in *Poplus euphratica* is an arginine residue, which was associated with a fitness advantage in saline environments in a genetic analysis of this species. We therefore mutated this residue to arginine to mimic the residue in *Poplus*. Our preliminary experiments were interesting therefore we made a number of other mutations of this residue to amino acids, alanine, phenylalanine and lysine. Only the mutation of this residue to arginine resulted in a strong positive effect, and that effect was on LiCl tolerance. There was a dramatic increase in growth in the LiCl containing medium at both high and low concentrations of LiCl (Figure 5 and Figure 6, Table 2). The effect was quite specific. A change to lysine, which has the same charge but is a different size, could not mimic this effect and even had a negative effect on LiCl tolerance. (It is however notable there was one slight improvement in NaCl tolerance at one high concentration of NaCl, though this was a small effect and only at one concentration of NaCl.) Other mutations to alanine or phenylalanine did show some improvements in salt tolerance that were not as dramatic. There were small but significant improvements in NaCl tolerance shown with the Y346 to alanine and the Y346 to phenylalanine mutation at lower NaCl concentrations. While not huge, these improvements at concentrations between 200 and 500 mM might be significant if they occurred in plants encountering salt challenges in the environment.

To understand how our mutagenesis experiments might be mediating their effects we examined a comparative analysis of two *Os*SOS1 structures. It revealed that the TM10-TM11 loop exhibits significant flexibility [59] (Figure 7). The location of the amino acid Y346 is within this same TM10-TM11 loop of the *A. thaliana* SOS1 protein (Figure 2 and Figure 3C). Multiple sequence alignment across a wide range of plant SOS1 proteins (Figure 2) indicated that, in most plants, a negatively charged amino acid is paired with a positively charged residue in this loop (Figure 2). As noted above, in salt-tolerant *Populus euphratica* SOS1 the corresponding position of Tyr346 in *A. thaliana* is occupied by arginine, and the same loop lacks any negatively charged residues. We replaced Tyr346 of *A. thaliana* SOS1 with arginine to explore its functional implications. Additionally, the cryo-EM structure of *Oryza sativa* SOS1 (*Os*SOS1) available in the PDB database shows that its TM10-TM11 loop contains an arginine residue preceded by an aspartate in the polypeptide sequence (Figure 2). Our results show that *At*SOS1 Y346R exhibits increased tolerance to Li^+^ while maintaining similar Na^+^ tolerance compared to wild-type AtSOS1. Since Tyr346 is not directly involved in metal ion coordination, this improved Li^+^ transport might result from an indirect effect of the substitution. In *A. thaliana*, Tyr346 is located near the upstream lysine residue (Lys343). Replacing Tyr346 with arginine increases the positive charge density at this position, potentially causing two effects. First, the positive charges of Lys343 and Arg346 may repel each other, pushing the arginine side chain away from lysine. Flexibility in this region could facilitate this effect. Interestingly, structural comparison shows that the corresponding loop in yeast plasma membrane Na^+^/H^+^ antiporter contains large amino acids such as tryptophan, phenylalanine and proline residues making it less flexible [65]. Second, Arg346 may interact with the membrane lipid head groups, possibly restricting the TM10-TM11 loop’s movement and widening the extracellular pore opening. We hypothesize that this structural adjustment could enhance Li^+^ transport specificity. Additionally, two hydrophobic residues are located just upstream to the substituted Tyr346Arg, isoleucine (Ile344) and alanine (Ala345). Previous studies suggest that Li^+^ may have a stronger affinity for hydrophobic surfaces [71]. Furthermore, Na^+^ has a larger hydration sphere than Li^+^, potentially requiring more protein interactions and coordination for effective transport. In contrast, the wider extracellular pore created by the Tyr346Arg substitution might better accommodate ions with smaller hydration radii, favoring Li^+^ transport. This is consistent with previous findings that ion hydration radii influence ion selectivity in ion channels [72].

While there was no improvement in NaCl tolerance with the Y346R mutation, there was a consistent minor effect with the Y346F mutation and a minor effect on NaCl tolerance with the Y346A mutation. As noted above with the L375I mutation, others found differing results [57]. Again, we suggest that the beneficial effect of changing these amino acids may be species-specific and not transferrable to the SOS1 protein expressed in our system. This makes these mutations less desirable target as a method of increasing salt tolerance in many plant species.

## 4. Conclusions

Overall, our results have shown several interesting novel findings. Most notable is the importance of the specific changes in amino acid Y346. There was a striking and dramatic effect on Na^+^ vs. Li^+^ tolerance when this residue was changed to Arg. The AY346R-containing protein showed a clear improvement in LiCl tolerance. Other mutations of amino acids showed minor improvements on NaCl tolerance, such as the mutations Y346F, A399V, Y346F and Y346A. While these changes were not huge, the possible effects of even a slight improvement in agricultural plant growth in arid and saline soils, makes future investigation of these effects in plants of interest in future experiments. It should be noted that our experiments were performed in a heterologous expression system. It is possible that some of these effects are altered in plant species. Therefore, verification in vivo in plants is suggested.

## 5. Materials and Methods

### 5.1. Materials

Restriction enzymes were purchased from New England Biolabs, Inc. (Mississauga ON, Canada). PWO DNA polymerase was purchased from Roche Applied Science (Roche Molecular Biochemicals, Mannheim, Germany). Other chemicals were of analytical grade and from Fisher Scientific (Ottawa, ON, Canada), Sigma or BDH (Toronto, ON, Canada).

### 5.2. Plasmids and Site-Directed Mutagenesis

The SOS1 expression plasmid used in this study (pREP41SOSsGFP) was described earlier [56] and is an expression plasmid that contains Arabidopsis thaliana SOS1 that expresses amino acids 1–481 containing the membrane transport domain of SOS1. Briefly, as described in our earlier publications [73,74] amino acids 1–481 of SOS1 were cloned into the plasmid pREP41GFP. The plasmid contains a Gly-Ala linker preceding GFP which contains the Ser65Thr mutation and has a NdeI site removed with a silent mutation. This “short” plasmid (referred to as SOS1short or SOS1s) construct expresses amino acids 1–481 of SOS1 fused to GFP through a Gly-Ala linker. We have earlier shown that it is functional and can restore salt tolerance to *S. pombe* with their own endogenous salt tolerance protein deleted [73,74] This system was used for all mutagenesis and expression experiments with SOS1. The plasmid pREP41sod2GFP [62] was used as a positive control and is a similar construct except expressing Sod2, the native Na^+^/H^+^ antiporter of *S. pombe*, which we have also characterized earlier in the same system [55,63,75]. Mutations to the pREP41SOSGFP plasmid containing the shortened SOS1 were by PCR amplification using synthetic oligonucleotides (See Table 2) as described earlier [75]. Mutations were designed to create or remove a restriction site that was used in screening mutants. DNA sequencing confirmed the accuracy of the mutations and fidelity of the resultant plasmid.

To examine the expression and activity of wild type and mutant SOS1 protein *S. pombe* with the sod2 gene disruption (sod2::ura4) was used to host transformations of yeast with wild-type and mutant GFP-tagged SOS1s [55,62]. Also, where indicated transformation was with the positive control of the same plasmid with a sod2 insert which is the positive control of the endogenous Na^+^/H^+^ antiporter of *S. pombe* [62,64]. The knockout strain was maintained on low sodium minimal KMAL medium or yeast extract adenine (YEA) [64,76]. For growth curves 2 × 10^6^ cells from wild type or SOS1 mutant containing *S. pombe* were used from an exponentially growing overnight cultures to inoculate 2.5 mL of fresh media liquid [55,56,64,75]. Cultures were grown at 30 °C in a rotary shaker. At the times indicated cells were harvested and growth at A_600_ was determined as we have described earlier [55,56,64,75]. Growth curve experiments were performed in triplicate at least three times. Growth on plates was determined in media supplemented with NaCl or LiCl at the concentrations indicated as we have described earlier [55,56,64,75]. Sod2 or SOS1s can return salt tolerance to *S. pombe* with its endogenous Na^+^/H^+^ exchanger removed. If a mutation renders these proteins non-functional salt tolerance is not restored [55,56,64,75].

To examine growth on solid phase media, a procedure independent of liquid phase growth was used as described earlier [55,56,64,75]. Serial dilutions of cells expressing wild type SOS1s, Sod2 or mutant SOS1s were inoculated onto agar with KMA medium containing leucine supplemented with either NaCl or LiCl at the indicated concentrations. The system has been used by us as described earlier to determine effects of mutations on the ability to mediate salt tolerance by both Sod2 and SOS1 [55,56,62,64,75,77].

*Western Blotting Analysis*—Western blot analysis of SOS1 compared the levels of protein expression in cell lysates from wild-type and mutant SOS1-containing yeast [63,78]. Cell lysates were made from transformed yeast cultures. The yeast cultures were pelleted at 3500× *g* for 10 min and then washed with double-distilled water. Next, they were then resuspended in a lysis buffer containing 50 mM Tris-HCl, 5 mM EDTA, pH 8.0, 1 mM dithiothreitol containing a protease inhibitor cocktail [79]. Cells were lysed by passage through an Emulsiflex homogenizer at a pressure of 25,000 psi. Non-lysed cells were pelleted at 3500× *g* for 5 min, and the supernatant was centrifuged at 14,000× *g* for 10 min. Enriched membranes in the supernatant were centrifuged at 100,000× *g* for 1 h, then resuspended in 50 mM Tris-Cl (pH 8.0), 150 mM NaCl, 1 mM EGTA, 5 mM EDTA, 1.0% (*v*/*v*) NP-40, 0.5% (*w*/*v*) deoxycholate, and 0.1% (*w*/*v*) SDS. Equal amounts of up to 25 μg of samples were resolved on 10% SDS/polyacrylamide gels [55,56,64,75]. For Western blotting, an anti-GFP polyclonal antibody (a gift from Dr. Luc Berthiaume, Dept. of Cell Biology, University of Alberta) was used as the primary antibody, as described earlier [55,56,75]. In this system, both Sod2 and SOS1s expressed proteins contained a C-terminally linked GFP protein, which we have used in Western blotting as described earlier [55,56,75]. The secondary antibody was Li-Cor goat anti-rabbit antibody conjugated to IRDye 680, detected on a Li-Cor Odyssey Imager [55,56,75].

*Sequence Alignment/Computational work*—Multiple sequence alignment was carried out by the MAFFT online server [80]. The representation of the multiple sequence alignment was performed using the ESPript server [81]. The protein representation was performed with Pymol [82].

## Figures and Tables

**Table 1:** Summary of the effect of mutations of SOS1s on the ability to confer salt tolerance compared to wild-type SOS1. Based on liquid phase assay. (**A**), growth in liquid media. (**B**), growth on solid media. − reduced growth, less than wild-type SOS1 at some time points at these concentrations; −,− greatly reduced growth compared with wild-type SOS1 at some time points at these concentrations; + growth slightly (but significantly) greater than wild-type SOS1 at some time points at these concentrations; ++, growth more improved over wild-type SOS1 by a greater amount or at more time points at the indicated concentrations; +++, growth greatly improved over wild-type SOS1; =, growth not significantly different from wild SOS1s at all, or almost all, time points at these concentrations; *, growth was reduced in LiCl free medium, ^ growth was increased in NaCl free medium.

A. Growth Effects in Liquid Media Relative to sSOS1.				
Mutant	Growth in 2–5 mM LiCl	Growth in 6, 8 LiCl mM	Growth in 200–500 mM NaCl	Growth in 600, 800 mM NaCl
E261K	−	+	−	=
A399V	−	=	+	=
E261KA399V	−	=	+	=
Y346A	−,−	=	+	=
Y346F	=	=	+, ^	=
Y346K	−,−	−	=	+
Y346R	+++	+++	=	=
L375I	=, *	=	−,−	−
Q362L	=, *	+, *	=	=
B. Growth Effects on Solid Media.				
Mutant	Growth in 2, 4 mM LiCl	Growth in 5, 8 LiCl mM	Growth in 200, 400 mM NaCl	Growth in 500, 800 mM NaCl
E261K	−	=	=	=
A399V	−	−	=	=
E261KA399V	−	−	=	=
Y346A	=	=	=	=
Y346F	=	=	+	+
Y346K	=	=	=	=
Y346R	++	+++	=	=
L375I	=	−,−	=	=
Q362L	=	=	=	=

**Table 2:** Oligonucleotides used for site-directed mutagenesis of SOS1. Mutated nucleotides are indicated in lowercase. Codons of changed amino acids are indicated in boldface type. Restriction sites introduced are underlined, and wherever indicated (-) a site was removed. Only the forward oligonucleotide of the pairs used for mutagenesis is shown.

Mutation	Oligonucleotide	Restriction Site
E261K	CAATGACACTGTtATA**aAG****G**ATTACTCTTACAATTGC	Psi1
Y346A	AGTGATAAGATTGCC**gca**CAAGGGAAcTCATGGCGATTTC	-EcoR1
Y346F	AGTGATAAGATTGCC**Ttc**CAAGGGAAcTCATGG	-EcoR1
Y346K	AGTGATAAGATTGCC**aag**CAAGGGAAcTCATGGCGATTTC	-EcoR1
Y346R	GATAAGATTGCC**cgC**CAAGGGAAcTCATGGCGATTTC	-EcoR1
L375I	GGAGTTCTATATCCA**aTT**cTgTGcagaTTTGGCTATGGTTTG	BsgI
Q362L	CTATACGTTTACATC**Ctc**CTcTCGCGTGTTGTTG	BseR1
A399V	GGTTTGAGGGGC**gTc**GTGGCTCTTGCAC	-Bts1
E261KA399V	CAATGACACTGTtATA**aA****G**ATTACTCTTACAATTGCGGTTTGAGGGGC**gTc**GTGGCTCTTGCAC	Psi and Bts1

## Data Availability

Data are available at https://demo.borealisdata.ca/dataset.xhtml?persistentId=doi:10.80240/FK2/LYTGJH, available 25 January 2025.

## Abbreviations

The following abbreviations are used in this manuscript:

SOS	Salt Overlay Sensitive
SOS1s	Salt Overlay Sensitive 1 protein shortened at the C-terminus
At	*Arabidopsis thaliana*
TM	Transmembrane
GFP	Green fluorescent protein
